# Beyond manikins: Virtual simulation training in a post‐COVID era

**DOI:** 10.1111/medu.15685

**Published:** 2025-04-03

**Authors:** Krista Hoek, Monique van Velzen, Christina Jaschinski, Elise Y. Sarton

## 1 WHAT PROBLEMS WERE ADDRESSED?

Anaesthesiology residents at Leiden University Medical Center regularly undergo simulation training with a full‐body manikin. This is a vital aspect of the clinical programme providing a stressful yet safe environment for effective critical resource management (CRM) training. Unfortunately, the COVID‐19 pandemic made real‐life simulations challenging due to organizational and preventive measures.

As a result, we explored asynchronous training opportunities utilizing a multiplayer virtual reality (VR) simulation. VR simulations can create personalized scenarios, facilitating differentiated learning through enhanced sensory immersion. VR offers full immersion with a high potential for visual effects, simultaneously allowing changes in patient characteristics such as sex, weight, external trauma and age, which is impossible with regular manikin training.

The three‐step approach involved (1) identifying user requirements, (2) developing a prototype and (3) assessing the project's viability and interest for expansion.

## 2 WHAT WAS TRIED?

To achieve our objective, a collaboration was set up between the LUMC and the Extended Reality lab at Saxion University of Applied Sciences. The latter institute is highly experienced with VR and XR as educational tools. A user‐centred design (UCD) approach was used, involving a multidisciplinary expert team throughout the development process to guide design decisions, incorporating failure modes and effects analysis (FMEA).^1^

1. *Identification of user requirements* included the identification of a work flow for all basic components of the VR application.
2. *Development of the prototype* with adaptable physiological features, allowing the trainer to modify them via a trainer dashboard in a multiplayer setting. This adjustment was deemed necessary to enable the adaptation of the scenario for any trainee with learning objectives in CRM, in line with the principles of cognitive load theory.
3. *Assessment* included the collection of qualitative and quantitative data through observations, group interviews, and questionnaires, evaluating user experience among around 15 residents and clinical educators in line with Kirkpatrick's first evaluation level. Key concepts assessed were overall enjoyment, interaction with the hardware, physical discomfort, presence, virtual embodiment, acceptability and perceived effectiveness.

## 3 WHAT LESSONS WERE LEARNED?

Prototype iterations were systematically tested in simulated settings, followed by expert debriefings and FMEA. Each cycle informed design enhancements, ensuring usability and training effectiveness. Adjustments continued iteratively until the prototype was deemed optimized. User evaluations revealed that medical interns and clinical educators believed in the potential and usefulness of the VR simulation as a learning tool for CRM.

Encountered challenges included onboarding in the virtual environment requiring participants to familiarize themselves with effective navigation within the virtual space.

Another challenge involved translating clinical needs into programming possibilities outlined by the developers. This continuous need for translation persisted between user requirements and technical capabilities. Integration of simulation with UCD and FMEA facilitated design and redesign of the prototype.

The anaesthesiology department provided full support, and a collaborative partnership was made with another university medical centre to facilitate further validation and broader implementation for other institutions enhancing scalability.

## AUTHOR CONTRIBUTIONS

**K. Hoek:** Conceptualization; investigation; funding acquisition; writing – original draft; methodology; validation; visualization; writing – review and editing; software; formal analysis; project administration. **M. van Velzen:** Conceptualization; supervision; writing – review and editing. **C. Jaschinski:** Conceptualization; investigation; project administration. **E.Y. Sarton:** Conceptualization; supervision; resources.

## CONFLICT OF INTEREST STATEMENT

None.

## ETHICS STATEMENT

The protocol was approved by the Institutional Science Committee, received a waiver to be performed by the Institutional Review Ethics Committee (METC Leiden the Hague Delft, Leiden, the Netherlands) and adhered to GCP regulations. Written informed consent was obtained from all participants. Participants received no financial compensation.

## Data Availability

The data that support the findings of this study are available from the corresponding author upon reasonable request.
